# MiR‐3116 sensitizes glioma cells to temozolomide by targeting FGFR1 and regulating the FGFR1/PI3K/AKT pathway

**DOI:** 10.1111/jcmm.15133

**Published:** 2020-03-17

**Authors:** Shiqi Kong, Yingxiao Cao, Xin Li, Zhenzhong Li, Yuling Xin, Yan Meng

**Affiliations:** ^1^ Department of Neurosurgery Xingtai People’s Hospital Xingtai China; ^2^ Department of Neurosurgery The First People's Hospital of Shenyang Shenyang China; ^3^ Department of Operating Room Xingtai People’s Hospital Xingtai China

**Keywords:** drug resistance, FGFR1, FGFR1/PI3K/AKT, miR‐3116, temozolomide

## Abstract

Glioma is a brain tumour that is often diagnosed, and temozolomide (TMZ) is a common chemotherapeutic drug used in glioma. Yet, resistance to TMZ is a chief hurdle towards curing the malignancy. The current work explores the pathways and involvement of miR‐3116 in the TMZ resistance. miR‐3116 and FGFR1 mRNA were quantified by real‐time PCR in malignant samples and cell lines. Appropriate assays were designed for apoptosis, viability, the ability to form colonies and reporter assays to study the effects of the miR‐3116 or FGFR1. The involvement of PI3K/AKT signalling was assessed using Western blotting. Tumorigenesis was evaluated in an appropriate xenograft mouse model in vivo. This work revealed that the levels of miR‐3116 dipped in samples resistant to TMZ, while increased miR‐3116 caused an inhibition of the tumour features mentioned above to hence augment TMZ sensitivity. miR‐3116 was found to target FGFR1. When FGFR1 was overexpressed, resistance to TMZ was augmented and reversed the sensitivity caused by miR‐3116. Our findings further confirmed PI3K/AKT signalling pathway is involved in this action. In conclusion, miR‐3116 sensitizes glioma cells to TMZ through FGFR1 downregulation and the PI3K/AKT pathway inactivation. Our results provide a strategy to overcome TMZ resistance in glioma treatment.

## 1 INTRODUCTION

Glioma accounts for 30% of brain tumours and 80% malignancies making it a ubiquitous malignancy of the brain.^1^, ^2^ While there have been developments to address this condition, the median survival is 12‐14 months for the high‐grade disease causing the prognosis.^3^ One approach is the use of chemotherapy (temozolomide: TMZ) plus surgery and radiotherapy. This approach, however, is challenged by TMZ resistance (inherent and acquired).^4^, ^5^ This calls for the addressing of this condition to boost the responses of the malignancy to TMZ.

MicroRNAs (MiRNAs) are the small endogenous non‐coding RNAs that could inhibit translation or degrade mRNA by base‐pairing to the 3′‐untranslated region (UTR) of targets to result in the inactivation of the latter.^6^, ^7^ Research has shown that miRNAs are oncogenes or tumour suppressors by targeting the cell cycle, cell division, apoptosis and angiogenesis in numerous human cancers.^8^ There is also proof of the functioning of miRNAs in the resistance to chemotherapeutic agents via their influence on the uptake, metabolism, targeting of the drug or influencing apoptosis or repair mechanisms or the cell cycle.^9^, ^10^ This is highlighted by an example of lowered TMZ resistance by boosting miR‐634 to target CYR61 to in turn affect the Raf‐ERK circuit in glioma.^11^ Oncogenesis is augmented by numerous tumour suppressor genes that are the target of miR‐21: that happens to be among the first discovered miRNAs in humans.^12^ Glioma cells were sensitized to TMZ by miR‐1268a overexpression that inhibited ABCC1.^13^ The functioning of miRNAs in chemoresistance is highlighted by these findings to hence propel one to explore the reason behind TMZ resistance in gliomas especially in the case of miR‐3116 that yet remains to be studied.

The fibroblast growth factor receptor (FGFR) family of receptor tyrosine kinases comprise of four factors: (FGFR1), FGFR2, FGFR3 and FGFR4 that mediate fibroblast growth factor (FGF) signals.^14^, ^15^ The modulation of cell division, growth, differentiation, survival and death by these FGFRs is known via the following pathways: phosphatidylinositide 3‐kinases (PI3K)/AKT, signal transducers and activators of transcription (STAT), Ras/mitogen‐activated protein kinase (MAPK), and phospholipase C gamma.^16^, ^17^, ^18^ The expression of these factors is boosted in the event of chromosomal aberrations such as translocation or amplification to hence make them worthy of perusal to function as targets for treating malignancies.^18^ Upon ligand binding, FGFRs dimerize and activate a complex downstream signalling, including MAPK, PI3K/AKT and STAT pathways.^19^ miR‐3116 is a miRNA that has not been fully studied. In the current study, we aimed to investigate the role of miR‐3116 in glioma.

This work shows a lowering of miR‐3116 in glioma samples displaying TMZ resistance. In addition, miR‐3116 was shown to target FGFR1 leading to PI3K/AKT inactivation. Our findings indicate miR‐3116/FGFR1/PI3K/AKT axis play a key role in TMZ resistance in glioma.

## 2 MATERIALS AND METHODS

### 2.1 Patient samples

Glioma patients scheduled for surgery at Xingtai People's Hospital from December 2014 to January 2017 were the source of tumour samples that were subjected to liquid nitrogen freezing and −80°C storage until use. TMZ was administered to all the patients post‐surgery. The allocation of patients was done into the two groups: response (n = 40) and non‐response (n = 40) on the basis of the Response Evaluation Criteria in Solid Tumors (RECIST) criteria. While the Ethics Committee of Xingtai People's Hospital issued an approval for this work, the patients issued informed written consent prior to the study.

### 2.2 Cell lines

The American Type Culture Collection was the source of human glioma cells U87 and U251. The generation of U87/TR and U251/TR lines showing resistance to TMZ was performed by exposing the initially sensitive culture to gradual increments of TMZ (final 400 μmol/L) over 6 months in our laboratory involving selection at each step and the subculture of resistant clones. The resistance was maintained by including 50 μmol/L TMZ (Sigma) during incubation. The medium used for all cell lines was plus 10% foetal bovine serum (Invitrogen) at 37°C and 5% CO_2_ in an incubator.

### 2.3 Transfection

GenePharma (Shanghai, China) was the source of MiR‐3116 (miR‐3116 mimic), anti‐miR‐3116 (miR‐3116 inhibitor, anti‐miR‐3116), miR‐NC and anti‐miR‐NC (corresponding scrambled controls). pcDNA3.1 vector (Invitrogen) was utilized for cloning FGFR1 cDNA and its subsequent overexpression (FGFR1). Lipofectamine 2000 reagent (Invitrogen) was utilized for transfection of 100 ng plasmids or 50 ng oligonucleotides on six‐well plates with cells in accordance with the prescribed protocols of the manufacturer.

### 2.4 Real‐time PCR

Read‐time PCR was performed as in previous studies.^20^, ^21^ Briefly, TRIzol reagent (Takara) was utilized to obtain total RNA from glioma tissues and cells. THUNDERBIRD SYBR^®^ qPCR Mix Kit (Sigma) was employed to assess the mRNA levels of miR‐3116 and FGFR1 on an ABI 7500 fast real‐time PCR system (Applied Biosystems). The 2^−ΔΔCT^ approach was employed to assess the relative levels with reference to the internal controls: U6 and β‐actin. The primers used in this study are listed as follows: miR‐3116, forward: 5′‐AATATTTGGTAATTGTTAGGGT‐3′, reverse: 5′‐TAACATCGTCCTTTAGCATT‐3′; U6, forward: 5′‐CTCGCTTCGGCAGCACA‐3′, reverse: 5′‐AACGCTTCACGAATTTGCGT‐3′; β‐actin, forward: 5′‐CAAAGGCCAACAGAGAGAAGAT‐3′, reverse: 5′‐TGAGACACACCATCACCAGAAT‐3′; and FGFR1, forward: 5′‐GGTTGACCGTTCTGGAAGC‐3′, reverse: 5′‐GCCCCGGTGCAGTAGATA‐3′.

### 2.5 Assay for drug resistance

Ninety‐six‐well plates were used to plate transfected U87/TR and U251/TR followed by the administration of indicated concentrations of TMZ for 48 hours. This was followed by the use of Cell Counting Kit‐8 (CCK‐8) to assess the viability of cells in accordance with the prescribed protocols of the manufacturer. Nonlinear regression was utilized to calculate IC_50_: half maximal inhibitory concentration. Prism V software (GraphPad Software Inc) was employed to fit the data on a sigmoidal dose‐response relation using the following association: Y = 100/(1 + 10^((LogIC_50_‐X)∗HillSlope)). X: log of TMZ dose. Y: normalized response, 100% to 0%, that dips with increasing X. logIC_50_: The unit is on par with X. Hill slope or slope factor lacks a unit.

### 2.6 Assay for apoptosis

Six‐well plates were used to plate transfected U87/TR and U251/TR that were exposed to TMZ as described above.^22^, ^23^ This was followed by FITC‐Annexin V and propidium iodide (PI; BD Biosciences) staining followed by flow cytometry (FACScan; BD Bioscience) analysis. The identification of cells was in accordance with the scale: Annexin V−/PI+: necrotic, Annexin V−/PI−: viable, Annexin V+/PI+: cells in late apoptosis and Annexin V+/PI−: cells in early apoptosis. Both cells in early and late apoptosis were counted for the apoptotic rate.

### 2.7 Assay for colony formation

Six‐well plates were used to plate transfected U87/TR and U251/TR that were exposed to TMZ as described above.^24^ Changing of the medium was done every 4 days post‐removal of TMZ. The 95% ethanol was utilized for fixation followed by 0.1% crystal violet staining after 14 days of incubation. Imaging and enumerating colonies with more than 50 cells were done.

### 2.8 Luciferase reporter assay

PCR amplification of the FGFR1 3′‐UTR region with the putative miR‐3116 binding sites (wild‐type, WT) and corresponding mutant sites (MUT) was done followed by subcloning into the Sac I and Hind III sites of the pmiRNA‐report firefly luciferase vector (Genechem). Lipofectamine 2000 (Invitrogen) was used to cotransfect U87/TR and U251/TR with the constructs so generated: FGFR1‐WT or FGFR1‐MUT and miR‐NC or miR‐3116. A dual‐luciferase assay kit (Promega) was employed to analyse the enzyme activity (firefly and Renilla luciferase) 48 hours post‐transfection. The relative intensities of both these enzymes were used to calculate the fold or relative activity.

### 2.9 Western blotting

Western blotting was performed as in previous reports.^25^, ^26^ Briefly, RIPA Buffer (Sigma) was utilized to lyse collected cells followed by analysing the concentration of proteins by a BCA Protein Assay Kit (Beyotime). SDS‐PAGE (10%) was run to resolve equal quantities of protein extracts (50 μg) followed by transfer to polyvinylidene fluoride membranes (Thermo Scientific). This was followed by the administration of primary antibodies overnight at 4°C. TBS‐T was utilized for washing thrice followed by the addition of HRP‐conjugated goat antimouse secondary antibody for 60 minutes at room temperature. Electrochemiluminescence was utilized to scan the membranes followed by imaging by a gel imaging system (Bio‐Rad Laboratories). The primary antibodies are listed as follows: FGFR1 (ab824, Abcam), p‐AKT (#4060), AKT (#9297), p‐mTOR (#1230), mTOR (#2972), cleaved caspase‐3 (#9661, Cell Signaling Technology) and β‐actin (A5441, Sigma).

### 2.10 Xenograft mouse model

Post‐issue of the Animal Care and Use Committee of Xingtai People's Hospital approval, the animal assays were within the framework of the national standard of laboratory animal use and care.

1. 5 × 10^6^ sh NC or sh FGFR1 U87 cells were injected into flank of Balb/C athymic nude (male; 6 weeks in age) mice (Shanghai Experiment Animal Center, China).
2. Subcutaneous injection of 5 × 10^6^ of U87 transfected with lenti‐miR‐NC or lenti‐miR‐3116 or uninfected was done into the right hind limb of Balb/C athymic nude (male; 6 weeks in age) mice (Shanghai Experiment Animal Center, China).

Intraperitoneal administration of 20 mg/kg TMZ was done one‐week post‐cell injection. A calliper was utilized to quantify the volume of a tumour every 2 days with the help of the following expression: volume = 1/2 × length × width^2^. Excision and weighing of tumours were done on day 19 post‐cell injection.

### 2.11 Statistical analysis

Mean ± SD was used to depict the quantitative data from minimum three independent experiments. One‐way ANOVA was employed to compare homoscedasticity between two groups, while homoscedastic data were assessed, using Student's *t* test. *P < .05, **P < .01 and ***P < .001 were considered significant.

## 3 RESULTS

### 3.1 Lowered miR‐3116 level in TMZ‐resistant glioma samples

**Figure 1:**
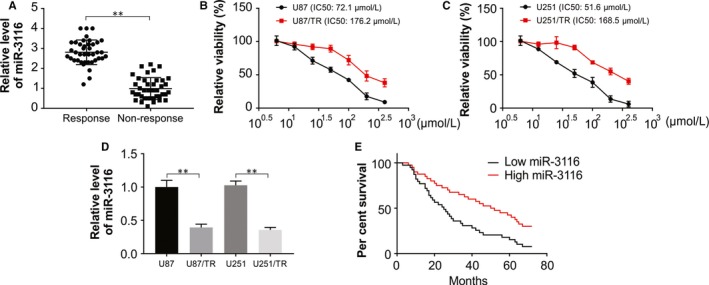
Lower miR‐3116 expression in TMZ‐resistant glioma and cell lines. A, Real‐time PCR analysis was performed to measure the expression level of miR‐3116 in patients showing response to TMZ and patients showing no response to TMZ. B, Cell survival was determined by CCK‐8 assay in U87 and U87/TR cells after treatment with different concentration of TMZ. C, Cell survival was determined by CCK‐8 assay in U251 and U251/TR cells after treatment with different concentration of TMZ. D, The expression level of miR‐3116 was detected in TMZ‐resistant glioma cells (U87/TR and U251/TR) and their parental cells (U87 and U251) by real‐time PCR. E, Glioma patients were classified into high miR‐3116 expression and low miR‐3116 expression groups. Kaplan‐Meier overall survival curves according to the relative miR‐3116 expression level. **P < .01

First, we aimed to assess the effect of miR‐3116 on the TMZ resistance‐glioma association involved observing the miR‐3116 level in patients exposed to TMZ. Patients resistant to TMZ displayed a conspicuous lowering of miR‐3116 against sensitive patients (Figure 1A). As described in the materials section, the TMZ‐resistant cells were generated in U87 and U251 cell lines, which were sensitive to TMZ normally. As shown in Figure 1B,C, the TMZ‐resistant cells U87/TR and U251/TR are resistant to TMZ‐induced growth inhibition, which was analysed by CCK‐8. This was followed by the analysis of miR‐3116 expression in both resistant cell lines and their parental cells. Our findings indicated that U87/TR and U251/TR displayed lowered miR‐3116 levels against their parental cell lines, respectively (Figure 1D). In addition, the survival time of patients was extended when the miR‐3116 level was high against patients with lower miR‐3116 level (Figure 1E). This is indicative of the role of lower miR‐3116 and resistance to chemotherapy (TMZ) in glioma samples that is finally associated with poor prognosis.

### 3.2 Increased TMZ sensitivity by miR‐3116 overexpression

**Figure 2:**
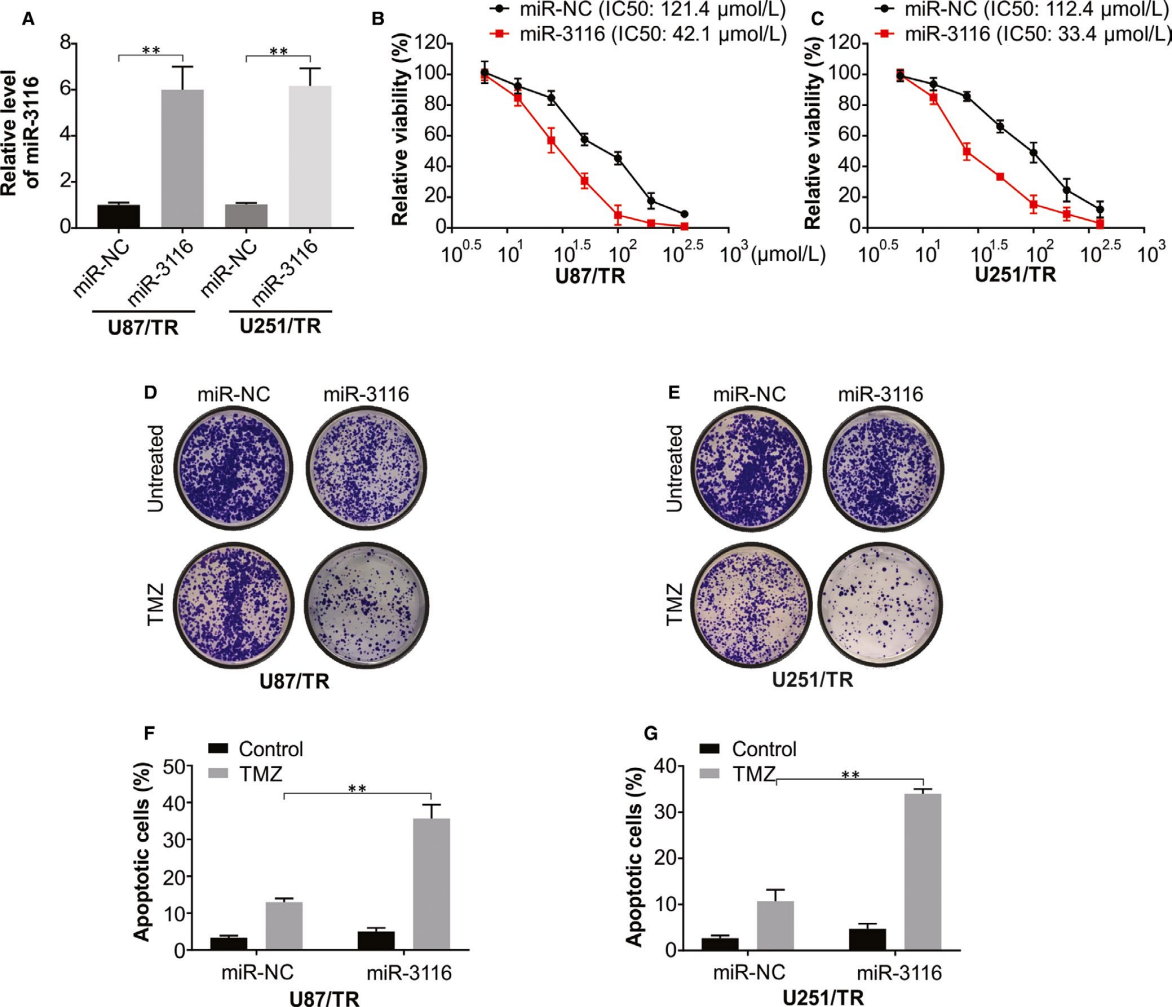
Overexpression of miR‐3116 increases the chemosensitivity to TMZ in TMZ‐resistant glioma cells. A, Real‐time PCR analysis was used to assess the transfection efficiency of miR‐3116 in U87/TR and U251/TR cells. B, CCK‐8 assay was applied to evaluate the effect of miR‐3116 on cell viability in U87/TR cells after treatment with different doses of TMZ. C, CCK‐8 assay was applied to evaluate the effect of miR‐3116 on cell viability in U251/TR cells after treatment with different doses of TMZ. D, Colony formation assay was carried out to determine the effect of miR‐3116 on the colony forming ability in U87/TR cells with or without TMZ treatment. E, Colony formation assay was carried out to determine the effect of miR‐3116 on the colony forming ability in U251/TR cells with or without TMZ treatment. F, Flow cytometry analysis was conducted to confirm the effect of miR‐3116 on apoptosis in U87/TR cells with or without TMZ treatment. F, Flow cytometry analysis was conducted to confirm the effect of miR‐3116 on apoptosis in U251/TR cells with or without TMZ treatment. **P < .01

Next, we investigated the regulatory function of miR‐3116 in the TMZ resistance in glioma via gain‐of‐function assays in glioma lines showing TMZ resistance. The transfection of miR‐3116 mimic resulted in obviously elevated miR‐3116 levels in the resistant cell lines (Figure 2A) that was also associated with an evident lowering of cell viability as shown by CCK‐8 assay against the miR‐NC cells (Figure 2B,C). Exposure of the resistant cells to TMZ when miR‐3116 was overexpression lowered the ability of the cells to form colonies (Figure 2D,E). This was followed by analysing the role of apoptosis in the sensitivity; U87/TR and U251/TR that were transfected with miR‐3116 displayed elevated apoptosis against miR‐NC‐ones (Figure 2F,G). This is indicative of the sensitization of glioma lines to TMZ when the miR‐3116 level was restored.

### 3.3 FGFR1 is a direct target of MiR‐3116 in resistant lines

**Figure 3:**
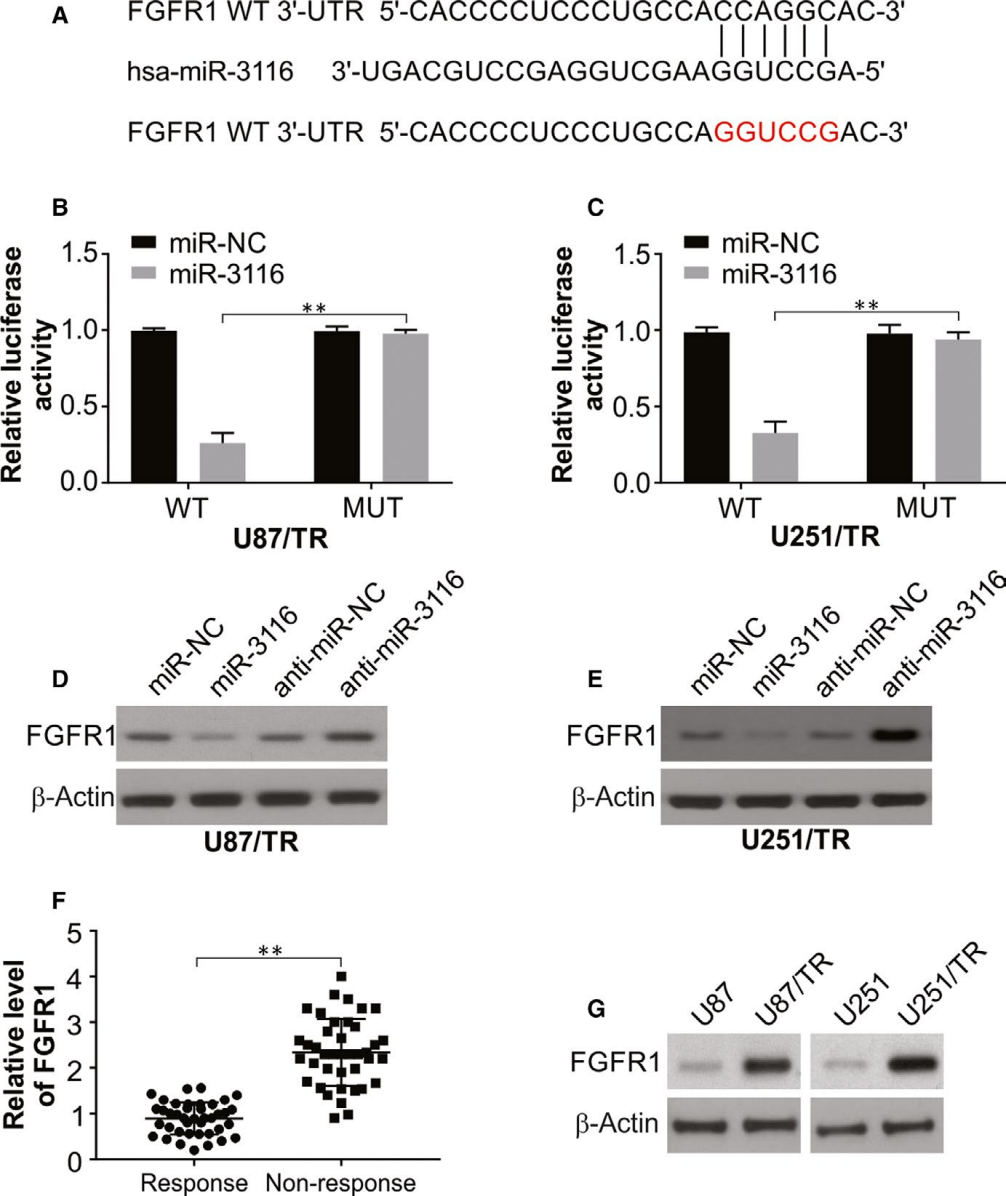
FGFR1 is identified as a direct target of miR‐3116 in TMZ‐resistant glioma cells. A, The predicted miR‐3116 target sequence in the 3′‐UTR of FGFR1, as well as the mutant containing altered nucleotides in the 3′‐UTR of FGFR1. B, Luciferase activity assay in U87/TR cells transfected with FGFR1‐WT or FGFR1‐MUT reporter and miR‐NC or miR‐3116. C, Luciferase activity assay in U251/TR cells transfected with FGFR1‐WT or FGFR1‐MUT reporter and miR‐NC or miR‐3116. D, Western blotting was performed to examine the effect of miR‐3116 overexpression or knockdown on FGFR1 protein level in U87/TR cells. E, Western blotting was performed to examine the effect of miR‐3116 overexpression or knockdown on FGFR1 protein level in U251/TR cells. F, Real‐time PCR was used to test the expression level of FGFR1 mRNA in tumour tissues from patients sensitive to TMZ and patients insensitive to TMZ. G, FGFR1 protein expression analysis in TMZ‐resistant glioma cells and corresponding parental glioma cells. **P < .01

As previously mentioned, the regulatory action of miRNAs occurs by binding to target genes that led us to assess the putative targets of the miRNA in this work using the bioinformatics algorithm miRcode (http://www.mircode.org/).^27^ This led to the identification of a region showing complementarity in the in 3′‐UTR of FGFR1 for miR‐3116 (Figure 3A) that was taken up further for analysis in this work. The generation of luciferase reporters containing WT or MUT FGFR1 binding sites was done to check this interaction. The resistant cells mentioned earlier displayed a conspicuous decrease in luciferase in the case of the WT sequence against the MUT sequence (Figure 3B,C). The effects were conspicuously checked using Western blotting that revealed that miR‐3116 overexpression caused an evident suppression of FGFR1, while miR‐3116 knockdown caused the reverse effect in the TMZ‐resistant cells (Figure 3D,E). Patient samples that were not responsive to TMZ displayed higher FGFR1 mRNA as against the TMZ responders (Figure 3F). In lieu of this was an up‐regulation of FGFR1 protein in the resistant lines generated against the parental glioma cells (Figure 3G). This is suggestive of the binding of FGFR1 3′‐UTR ‐ miR‐3116 to cause inhibition of the former by the latter.

### 3.4 Overexpressed FGFR1 attenuates TMZ sensitivity mediated by miR‐3116 glioma

**Figure 4:**
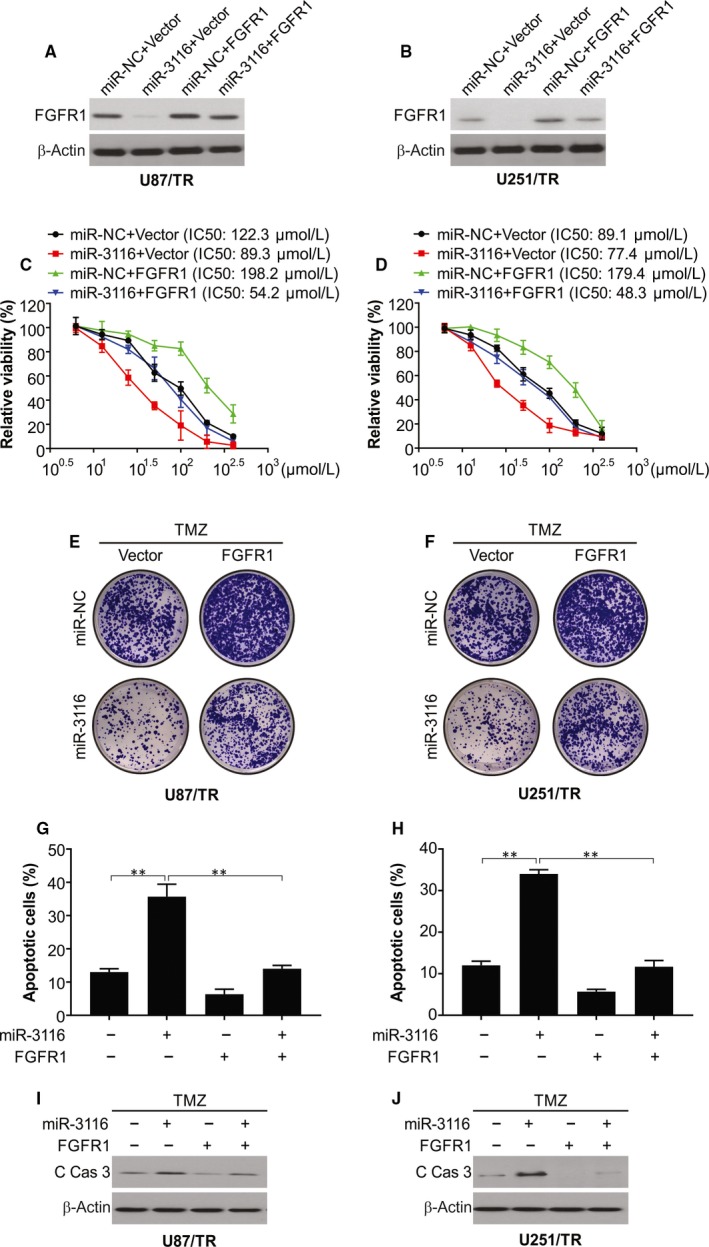
FGFR1 overexpression reversed miR‐3116‐mediated TMZ sensitivity in TMZ‐resistant glioma cells. A, U87/TR cells were transfected with miR‐NC or miR‐3116, together with or without FGFR1‐overexpressing plasmid, followed by Western blotting of FGFR1 protein level. B, U251/TR cells were transfected with miR‐NC or miR‐3116, together with or without FGFR1‐overexpressing plasmid, followed by Western blotting of FGFR1 protein level. C, Transfected U87/TR cells were treated with a serial dose of TMZ, and then, the cell viability of TMZ was monitored by CCK‐8 assay. D, Transfected U251/TR cells were treated with a serial dose of TMZ, and then, the cell viability of TMZ was monitored by CCK‐8 assay. E, Transfected U87/TR cells were treated with indicated dose of TMZ, and then, the colony forming ability was identified at day 14. F, Transfected U251/TR cells were treated with indicated dose of TMZ, and then, the colony forming ability was identified at day 14. G, Apoptosis was analysed in TMZ‐resistant U87/TR cells and parental U87 cells. H, Apoptosis was analysed in TMZ‐resistant U251/TR cells and parental U251 cells. I, Cleaved caspase‐3 was analysed in TMZ‐resistant U87/TR cells and parental U87 cells. J, Cleaved caspase‐3 was analysed in TMZ‐resistant U251/TR cells and parental U251 cells. **P < .01

The next step was transfecting the generated resistant lines with miR‐NC or miR‐3116 in the presence or absence of overexpressed FGFR1 and TMZ administration. pcDNA‐FGFR1 transfection caused the restoration of FGFR1 in lieu of expectations that reversed the FGFR1 suppression caused by miR‐3116 (Figure 4A,B). Functionally, this FGFR1 overexpression displayed reverse effect to that of miR‐3116 previously reported in terms of cell viability (Figure 4C,D), ability to form colonies (Figure 4E,F) and apoptosis (Figure 4G,H). This was also found to abolish the compromised cell viability and IC_50_ induced by miR‐3116 as shown by CCK‐8 assay (Figure 4C,D). The increase in FGFR1 also reversed the compromised ability to form colonies (Figure 4E,F). The apoptosis induced by miR‐3116 was also lowered by the restored levels of FGFR1 post‐TMZ treatment as shown by flow cytometry and Western blotting (Figure 4G‐J). All these FGFR1 associated effects were significantly depressed when miR‐3116 was cotransfected in terms of higher cell viability and IC_50_ (Figure 4C,D), higher ability to form colonies (Figure 4E,F), and lowered apoptosis (Figure 4G‐J). This is indicative of FGFR1 down‐regulated by miR‐3116 to augment the sensitivity of glioma lines to TMZ.

### 3.5 miR‐3116 inhibited PI3K/AKT signalling in TMZ‐resistant glioma cells by targeting FGFR1

**Figure 5:**
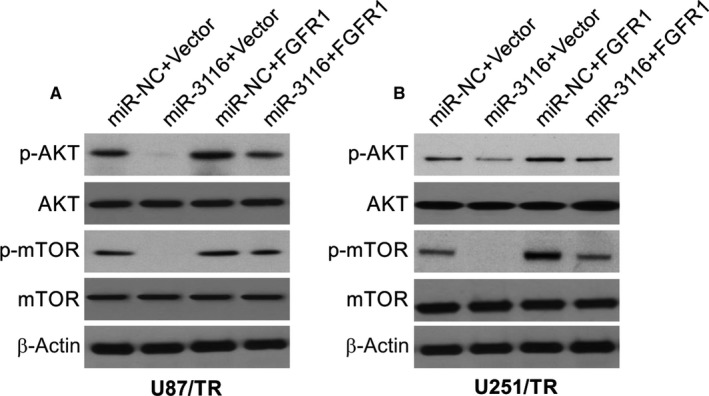
miR‐3116‐mediated inactivation of PI3K/AKT signalling was abated by FGFR1 up‐regulation. A, Western blotting was used to assess the effects of miR‐3116 or FGFR1 on the protein levels of p‐AKT and p‐mTOR in U87/TR cells. B, Western blotting was used to assess the effects of miR‐3116 or FGFR1 on the protein levels of p‐AKT and p‐mTOR in U251/TR cells

Progression of the glioma, clinical‐grade diagnosis and prognosis are influenced by PI3K/AKT signalling.^28^ A loss of control in FGF/FGFR pathways has been associated with the advancement of cancer according to research.^19^, ^29^ This oncogenesis is mediated by several target molecules such as PI3K/AKT and others already mentioned above. The activation of PI3K/AKT by FGFR1 was found to augment epithelial‐to‐mesenchymal transition and hence, metastasis in prostate cancer was shown in an earlier study.^30^ This led us to explore the role of miR‐3116 on FGFR1 and PI3K/AKT in the glioma cells under study. In lieu of expectations, overexpressed miR‐3116 caused an evident inhibition of AKT phosphorylation and mTOR phosphorylation with conspicuous reversal when the levels of FGFR1 were increased (Figure 5A,B). There was increased phosphorylation of AKT and mTOR upon up‐regulating FGFR1 that was reversed with miR‐3116 cotransfection (Figure 5A,B). The above data indicate that miR‐3116 inactivates the PI3K/AKT pathway by targeting FGFR1, which subsequently increased the sensitivity of the glioma line to TMZ.

### 3.6 miR‐3116 was anti‐tumour in xenograft mice while augmenting TMZ‐mediated anti‐tumour activities

**Figure 6:**
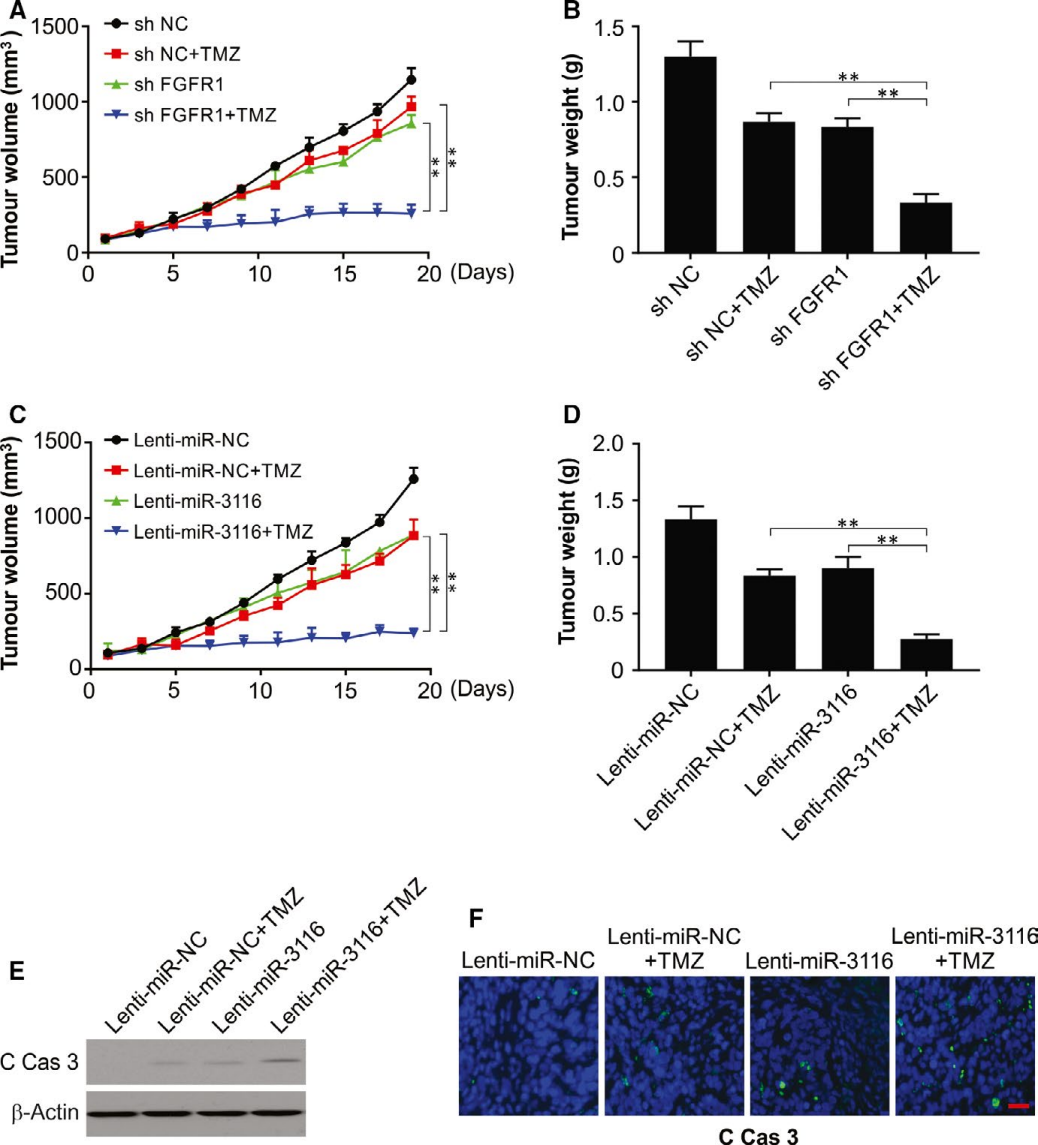
FGFR1 knockdown or miR‐3116 overexpression enhanced TMZ‐mediated anti‐tumour effect in U87/TR cell–derived glioma xenograft models. A and B, U87/TR cells infected with sh Con or sh FGFR1 were injected subcutaneously into mice. One week later, TMZ was administered intraperitoneally into mice daily at a dose of 20 mg/kg bodyweight for a total of 10 d. Tumour volume was analysed every two days. C and D, U87/TR cells infected with lenti‐miR‐Con or lenti‐miR‐3116 lentiviruses were injected subcutaneously into mice. One week later, TMZ was administered intraperitoneally into mice daily at a dose of 20 mg/kg bodyweight for a total of 10 d. Tumour volume was analysed every 2 days. E, The levels of cleaved caspase‐3 in randomly selected tumours were analysed by Western blotting. F, Paraffin‐embedded sections of tumour tissues from mice treated as in (C) were analysed by IF staining. **P < .01

To test the function of miR‐3116 in vivo, we used xenograft mouse model. As shown in Figure 6A,B, TMZ reduced tumour growth significantly in FGFR1 knockdown U87/TR tumours. In addition, the combination of TMZ and miR‐3116 reduced tumours significantly than single treatment in U87/TR tumours (Figure 6C,D). The levels of cleaved caspase‐3 were found to be higher in the combination treatment groups, which were analysed by Western blotting and IF staining (Figure 6E,F). The above data demonstrated that miR‐3116 or FGFR1 knockdown sensitized tumours to TMZ in vivo.

## 4 DISCUSSION

TMZ is the major treatment for patients with glioma that is challenged by resistance in patients.^31^, ^32^, ^33^ This requires an augmentation of drug efficiency at a clinical level by assessing the mechanisms associated with the resistance patterns.^33^ The past few years have seen the exhaustive analysis of the oncogenic potential of miRNAs.^34^, ^35^ The involvement of these molecules in resistance to chemotherapy has been documented via different pathways.^36^ This propelled us to explore the involvement of miR‐3116 in the resistance towards TMZ in the case of glioma samples.

In the current study, glioma patient samples and cell lines resistant to TMZ were found to display low levels of miR‐3116. When miR‐3116 was overexpressed in cell lines engineered to be resistant to TMZ, the ability of glioma cells to form colonies and remain viable was lowered, while apoptosis was increased. This is suggestive of boosted sensitization of glioma to TMZ by miR‐3116. The use of bioinformatics and reporter assays allowed for the finding that miR‐3116 directly targets FGFR1 and caused the inhibition of the latter.

Glioma has shown amplified levels of many FGFR molecules among which patients displaying poor responses showed increased FGFR1 that was not seen in chemotherapy‐responsive patients.^37^, ^38^ The various downstream signalling targets of this molecule such as PI3K/AKT have already been mentioned above. This work showed augmented levels of FGFR1 in the resistant lines that were in lieu of a recent study that showed similar up‐regulation in U87/TR cells.^39^ FGFR1 has also been shown to be oncogenic in the case of glioma by modulating the ability of cells to divide, migrate and invade.^40^ This work showed a direct interaction between miR‐3116 and FGFR1 in which the latter was inhibited by the former. Exogenous expression of FGFR1 caused an increase of our TMZ‐resistant cell lines to form colonies and remain viable while lowering apoptosis that is suggestive of the role of FGFR1 TMZ resistance in glioma. The increased TMZ sensitization caused by miR‐3116 was evidently nulled by the exogenous FGFR1 increase in the cell lines under study. This is illustrative of FGFR1 suppression by miR‐3116 to allow cells to be targeted by TMZ.

The involvement of PI3K/AKT signalling in glioma is known in terms of regulating the ability of cells to divide, migrate, invade and undergo angiogenesis.^41^, ^42^ Oncogenesis caused by FGFR1 was earlier shown to be associated with PI3K/AKT signalling pathway.^43^ Our findings showed inhibition of PI3K/AKT pathway by overexpressed miR‐3116, while overexpressed FGFR1 showed opposite results in addition to reversing the suppression of PI3K/AKT signalling caused by miR‐3116. The putative involvement of PI3K/AKT and its inactivation is the means by which miR‐3116 allows for glioma cells to be targeted by TMZ via FGFR1 as a target.

There are some limitations in this study. First, AKT inhibitors should be used to inhibit FGFR1‐ or miR‐3116 inhibitor‐mediated tumour cell growth in vitro and in vivo. Second, TCGA data should be provided to analyse the levels of miR‐3116 and FGFR1 in TMZ response and non‐response patients with glioma. Third, in the current study, only xenograft mouse model was used. Organoids, PDX mouse and orthotopic mouse model will be considered to investigate the role of miR‐3116 and FGFR1 in glioma in the future.

In conclusion, TMZ‐resistant glioma was found to show lowered miR‐3116 which accounts for the resistance. This work also highlighted that the reestablishment of this miR‐3116 caused an inhibition of FGFR1 and PI3K/AKT to, hence, allow glioma cells to be acted upon by TMZ. Our results provide a novel strategy for combining miR‐3116 with TMZ to effectively treat gliomas.

## CONFLICT OF INTEREST

The authors declare that there are no conflicts of interest.

## AUTHOR CONTRIBUTIONS

SK and ZL conceived and designed the study. SK, YC, XL and ZL performed the experiments and analysed data. SK, ZL, YX and YM organized data and wrote the manuscript. All authors read and approved the final version of the manuscript.

## Data Availability

The data that support the findings of this study are available from the corresponding author upon reasonable request.
